# Tumor-infiltrating B cells producing antitumor active immunoglobulins in resected HCC prolong patient survival

**DOI:** 10.18632/oncotarget.20238

**Published:** 2017-08-09

**Authors:** Stefan M. Brunner, Timo Itzel, Christoph Rubner, Rebecca Kesselring, Eva Griesshammer, Matthias Evert, Andreas Teufel, Hans J. Schlitt, Stefan Fichtner-Feigl

**Affiliations:** ^1^ Department of Surgery, University Medical Center Regensburg, Regensburg, Germany; ^2^ Institute of Pathology, University Medical Center Regensburg, Regensburg, Germany; ^3^ Department of Internal Medicine I, University Medical Center Regensburg, Regensburg, Germany; ^4^ Department of General and Visceral Surgery, University Medical Center Freiburg, Freiburg, Germany

**Keywords:** B cell, immune infiltrate, tumor microenvironment, gene expression, immunoglobulin

## Abstract

**Background & Aims:**

The immunological microenvironment of HCC influences patient outcome, however, the role of B cells remains unclear. This study investigated effects of local B-cell infiltration in HCC cohorts on patient survival and immunological and molecular tumor microenvironment.

**Results:**

Unsupervised gene expression analysis of full cancer transcriptomes (N=2158) revealed a highly co-regulated immunological cluster in HCC that mainly contained immunoglobulin fragments. More specifically, in an independent patient cohort (N=242) that compares HCC with non tumorous liver tissue high expression of these B-cell associated genes was associated with better patient outcome (P=0.0149). Conclusively, the immunohistochemical analysis of another independent cohort of resected HCCs (N=119) demonstrated that infiltration of HCCs by CD20^+^ cells (P=0.004) and CD79a^+^ cells (P=0.038) at the infiltrative margin were associated with prolonged patient survival. Further, the immunoglobulin fragments that were identified in the gene expression analysis were detected at high levels in patients with dense B-cell infiltration.

**Methods:**

Gene expression of 2 independent HCC tissue databases was compared using microarrays. Additionally, tissue of resected HCCs was stained for CD20, CD79a and immunoglobulins and analysed for the respective cell numbers separately for tumor, infiltrative margin and distant liver stroma. These findings were correlated with clinical data and patient outcome.

**Conclusions:**

Infiltration of HCCs by B cells is associated with prolonged patient survival. Further, a distinct B-cell like immunoglobulin profile of HCCs was identified that goes along with better patient outcome. We suggest that B cells contribute to local tumor control by secreting increased levels of immunoglobulins with antitumor activity.

## INTRODUCTION

Hepatocellular carcinoma (HCC) is the most frequent liver cancer entity worldwide with an increasing incidence [1, 2]. Potential therapy regimens include surgical resection or liver transplantation, local treatment methods like transarterial chemoembolisation or percutanous ablation, radiation or medical therapy [3]. Though, for the majority of HCC patients outcome remains poor due to tumor recurrence and the fact that many patients present with disease that is too advanced for curative surgery or liver transplantation [4]. This illustrates an urgent need to better understand the immunopathogenesis of HCC to develop new therapies [5].

Recently, our group has demonstrated that tumor-infiltrating, interleukin-33-producing CD8^+^ T cells in resected HCC prolong patient survival [6]. These CD8^+^ T cells have been characterized as highly effective, cytotoxically active CD8^+^CD62L^-^KLRG1^+^CD107a^+^ effector-memory cells producing IL-33 [6]. These tumor antigen specific cytotoxic CD8^+^ T cells primed by antigen-presenting dendritic cells represent the key cell subset in most immunotherapy trials in HCC that have demonstrated positive influence on patient survival [7, 8]. In contrast to these conclusive results that illustrate the beneficial influence of T cells on HCC outcome the studies investigating B-cell effects on HCC patient survival are contradictory.

It has been shown that atypical memory B cells that infiltrate HCCs especially through functional interaction with T cells are associated with better outcome [9, 10]. This B-cell subtype in combination with CD8^+^ cells was also identified to promote favorable prognosis in ovarian cancer [11]. In this study this was uncoupled with changes of antibody levels so the antitumor effect is thought to be due to B-cell support of cytotoxic T cells [11]. Contrarily, other B-cell subsets like CXCR3^+^ B cells that constitute approximately 45% of tumor-infiltrating B cells were shown to positively correlate with early recurrence of HCC and induce a protumorigenic activity of tumor-associated macrophages [12]. Further studies demonstrated that a higher percentage of the recently discovered regulatory B cells in serum, which were also detected at increased numbers in infiltrative tumor margins of human HCC specimens, were correlated with more advanced tumors [13]. In a SCID mouse model, these regulatory B cells promoted HCC growth and invasiveness by directly interacting with liver cancer cells through the CD40/CD154 signaling pathway [13]. Similarly it was reported, that IL-10 producing regulatory B cells in a combination with dendritic cell dysfunction might be responsible for HCC progression [14]. Additional studies did not show significant influence of B cells on HCC outcome [15]. Altogether, these varying results underline the importance of improved functional understanding of different phenotypes of tumor-infiltrating B cells and their mechanisms of antitumor activity [16].

Therefore this study was performed under the hypothesis that infiltration of HCCs by B cells is beneficial for patient survival after resection of HCC by direct antitumor effects through immunoglobulins. Further, we aimed to investigate if a distinct B-cell like immunoglobulin profile is associated with improved patient outcome in independent HCC and other cancer cohorts.

## RESULTS

### Bioinformatics co-expression analysis of large-scale oncogenetic microarray data identifies network of immunoglobulin fragments to be relevant in HCC

**Figure 1:**
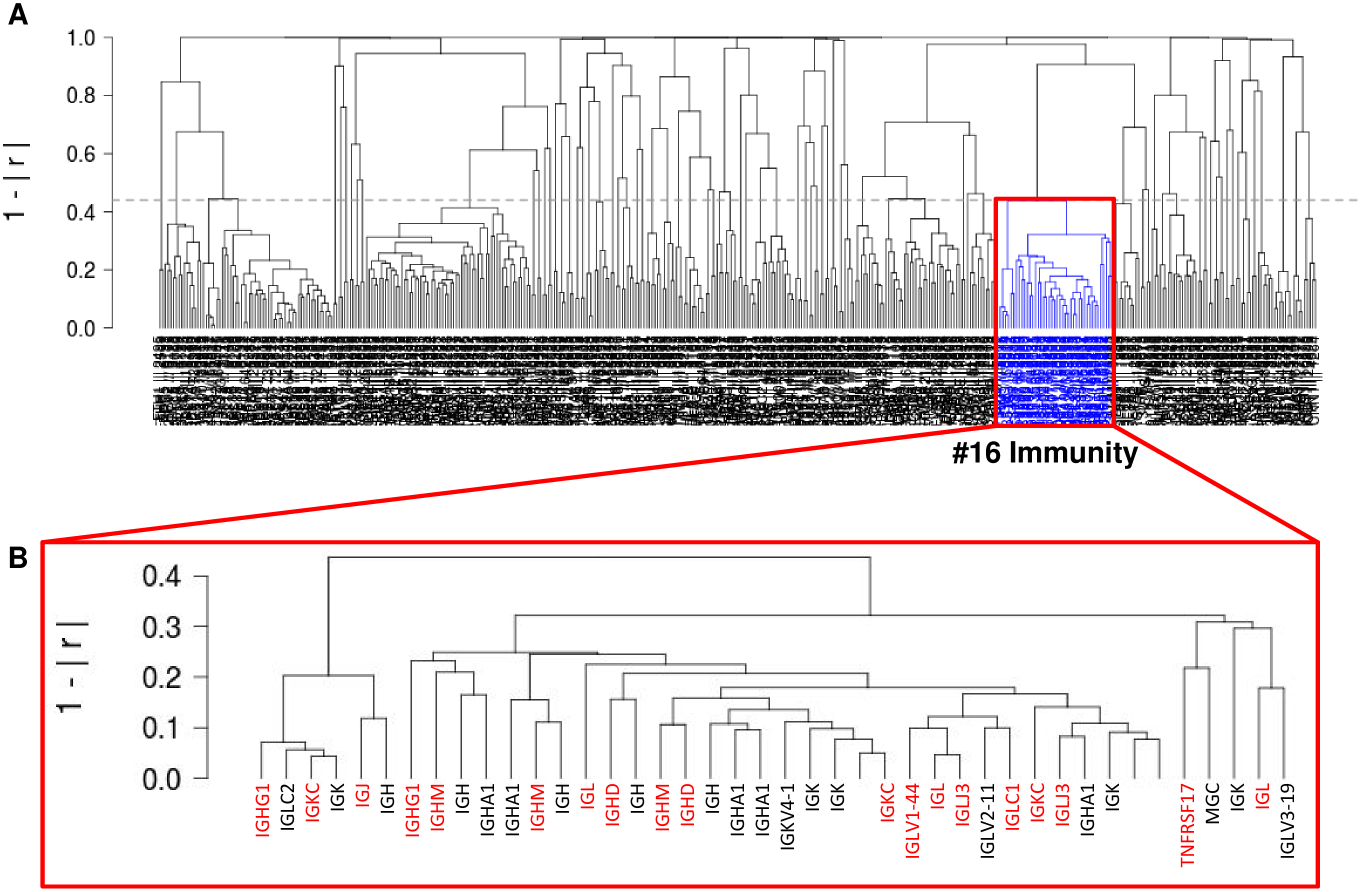
Unsupervised gene expression analysis of 2158 tumor samples containing 163 different tumor entities (GSE14520; NCBI-GEO) **(A)** A co-regulated 42 gene-containing cluster (#16) was identified (CC: 0.56-0.96) that was related with immunity and immunological events. **(B)** Most genes of this cluster coded for immunoglobulins or immunoglobulin fragments.

In a continued effort to identify networks of co-regulated genes we had previously analyzed a cohort of 2158 tumor samples and corresponding microarray data containing 163 different tumor entities [17]. As one of the largest co-regulated networks a 42 gene-containing cluster (#16) was identified (CC: 0.56-0.96) that was related with immunity and immunological events (Figure 1A). More specifically, most of these genes coded for immunoglobulins or fragments of immunoglobulins (Figure 1B).

**Figure 2:**
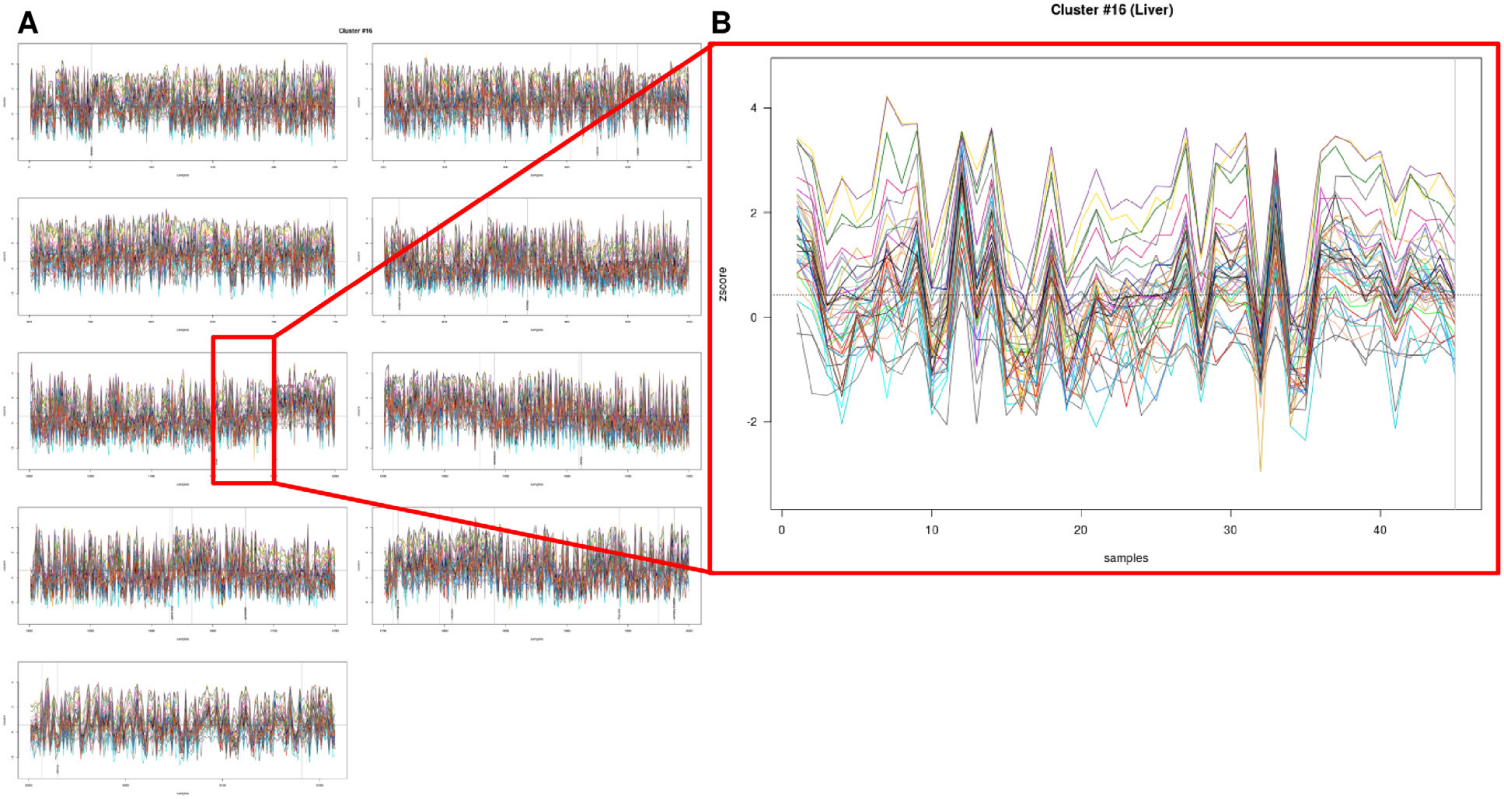
Detailed gene expression analysis of 2158 tumor samples with regards to HCC tissue (45 samples, 2.1% of overall samples) **(A** and **B)** The identified network of genes representing immunoglobulin fragments (cluster #16) showed a robust and stable co-expression in HCC tissue.

Given the hypothesis, that co-regulated networks functionally interact, these subnetworks should not only be detected in overall analysis but also be relevant within the individual tumor entities. Indeed, the identified network of genes representing immunoglobulin fragments (cluster #16) showed a robust and stable co-expression in HCC tissue (45 samples, 2.1% of overall samples; Figure 2A and 2B).

### Overexpression of immunoglobulins in independent HCC cohort is associated with prolonged patient survival

**Figure 3:**
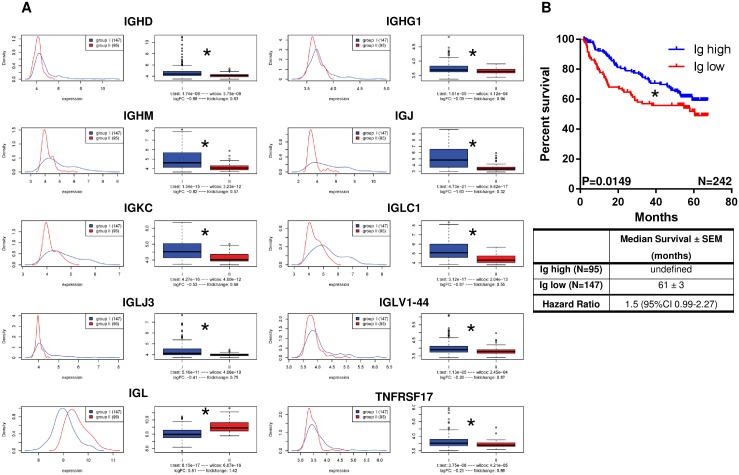
Overexpression of immunoglobulins in independent HCC cohort (GSE14520; N=242 HCC patients) is associated with prolonged patient survival **(A)** 10 out of the 42 genes that were co-regulated in the unsupervised gene expression analysis were also co-regulated in this independent HCC cohort. **(B)** Patients with significant overexpression of these genes in HCC tissue when compared to normal liver tissue survived significantly longer than patients with low expression of immunoglubulins (P=0.0149; Hazard Ratio 1.5 (95%CI 0.99-2.27).

To independently confirm the relevance of immunoglobulin expression in HCC tissue the genes identified in the large-scale database were tested in an independent HCC database (´GSE14520`) that contains microarray and survival data of 242 HCC patients [19]. 10 out of the 42 genes that were initially identified to be co-regulated were also relevant in this independent HCC cohort (Figure 3A). Moreover, patients who showed significant overexpression of these genes in HCC tissue when compared to normal liver tissue survived significantly longer than patients with low immunoglubulin expression (P=0.0149; Hazard Ratio 1.5 (95%CI 0.99-2.27); Figure 3A and 3B).

### Localized infiltration of CD20^+^ cells in the infiltrative margin is associated with increased patient survival after HCC resection

To explore if these effects of immunoglobulin expression on patient survival are attended by influences of B cells on outcome another independent patient cohort after surgical HCC resection was analyzed by immunohistochemistry. This collective consisted of 119 patients (26 female, 22%; 93 male, 78%) in whom resection was performed for HCC with a median age at operation of 65 years (IQR 58 – 71) and an overall median survival time of 47 ± 9 months after resection of the HCC with a 5-year survival rate of 39.9% [6].

**Figure 4:**
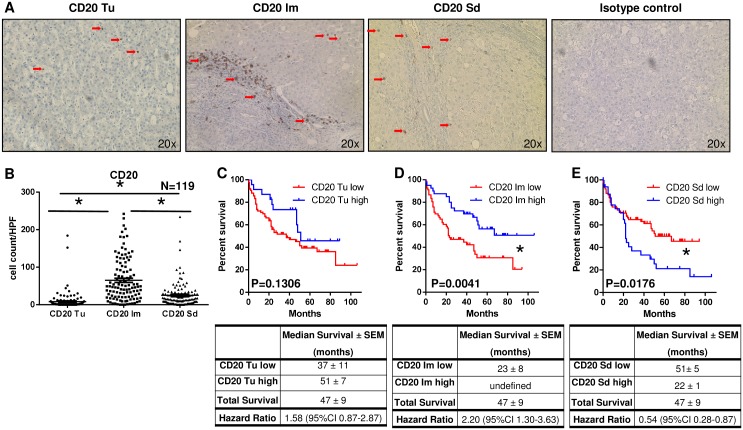
Localized infiltration of CD20^+^ cells within the infiltrative margin is associated with increased patient survival **(A)** Representative immunohistochemical stainings for CD20^+^ cells (brown) separately for tumor (Tu), infiltrative margin (Im), distant, normal liver stroma (Sd) and isotype control (red arrows indicate positive cells). **(B)** Most CD20^+^ cells were localized in the Im region around the tumor (65 ± 5 cells/HPF) which was significantly more than in the Tu (8 ± 2 cells/HPF; P<0.0001) and in the Sd area (25 ± 3 cells/HPF; P<0.0001). **(C)** Patients with high CD20^+^ cell numbers in the Tu showed a trend to better survival than patients with low CD20^+^ cell infiltration (P=0.1306). **(D)** Increased patient survival was noted in patients with high CD20^+^ cell numbers in the Im region compared to patients with low infiltration by CD20^+^ cells (P=0.0041). **(E)** In the Sd area patients with low CD20^+^ cell numbers survived significantly longer than patients with high CD20^+^-cell-infiltration (P=0.0176).

Immunohistochemical staining for CD20^+^ cells revealed that most of these cells were localized in the Im region around the tumor (65 ± 5 cells/HPF) which was significantly more than in the Tu (8 ± 2 cells/HPF; P<0.001) and in the Sd area (25 ± 3 cells/HPF; P<0.001; Figure 4A and 4B).

For survival analysis patients were divided into two groups, one with high and one with low infiltration by CD20^+^ cells. A trend but no significant survival differences based on CD20^+^ cell infiltration were detected in the Tu region (P=0.1306; Figure 4C). In the Im around the tumor significantly increased patient survival was noted in patients with high CD20^+^ cells numbers compared to patients with low infiltration by these cells (P=0.0041; Figure 4D). In contrast, in the Sd area patients with low CD20^+^ cell numbers survived significantly longer than patients with high CD20^+^-cell-infiltration (P=0.0176; Figure 4E).

In addition, high or low infiltration by CD20^+^ cells in the Im around the tumor did not correlate with clinical factors like gender, age, T stage, bridging therapy and also not with presence of hepatitis B or C. However, there was a significant correlation of cirrhosis with higher levels of infiltrating CD20^+^ cells in the infiltration margin (Supplementary Table 1).

We further tested, if CD20^+^ cells in the Tu and the Im region correlated with IL-33^+^ and CD8^+^ cells, which had significant impact on patient survival in previous experiments. Indeed, CD20^+^ cells in the Tu correlated with CD8^+^ cells in the Tu (P<0.001; Supplementary Figure 1).

### Localized infiltration of CD79a^+^ cells in the infiltrative margin is associated with increased patient survival after HCC resection

To confirm the results for CD20^+^ cells, HCC samples were analyzed by immunohistochemistry for CD79a, another pan-B-cell marker that is expressed throughout B-cell-development from very early precursor cells until maturation into plasma cells [20].

**Figure 5:**
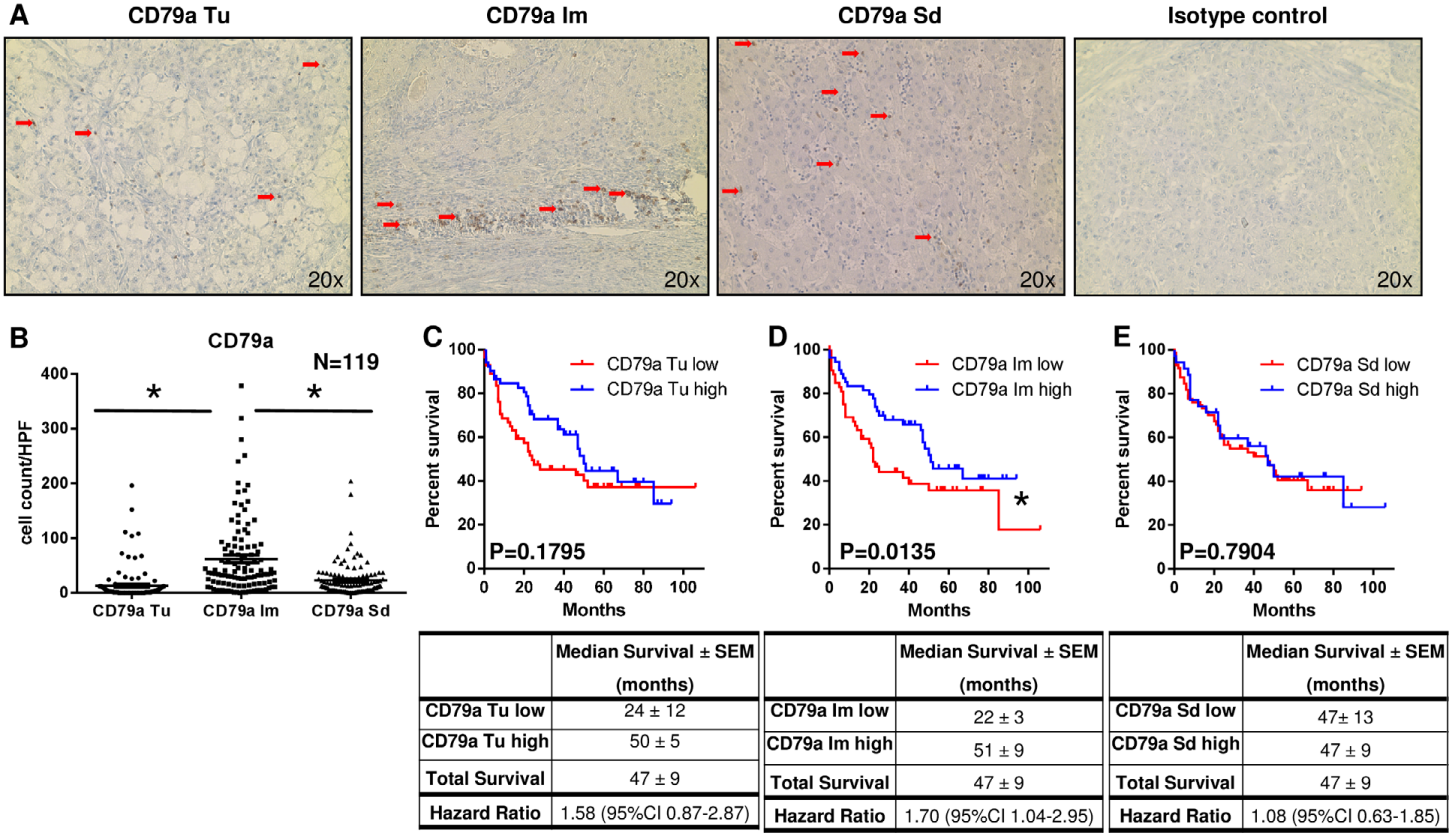
Localized infiltration of CD79a^+^ cells within the infiltrative margin is associated with increased patient survival **(A)** Representative immunohistochemical stainings for CD79a^+^ cells (brown) separately for tumor (Tu), infiltrative margin (Im), distant, normal liver stroma (Sd) and isotype control (red arrows indicate positive cells). **(B)** Most CD79a^+^ cells were localized in the Im region around the tumor (62 ± 7 cells/HPF) which was significantly more than in the Tu (13 ± 3 cells/HPF; P<0.0001) and in the Sd area (23 ± 3 cells/HPF; P<0.0001). **(C)** Patients with high CD79a^+^ cell numbers in the Tu showed a trend to better survival than patients with low CD79a^+^ cell infiltration (P=0.1795). **(D)** Increased patient survival was noted in patients with high CD79a^+^ cell numbers in the Im region compared to patients with low infiltration by CD79a^+^ cells (P=0.0135). **(E)** In the Sd area patients with low and high CD79a^+^ cell numbers showed no survival differences (P=0.7904).

In this analysis the highest CD79a^+^ cell number was detected in the Im region (62 ± 7 cells/HPF) which was significantly more than in the Tu (13 ± 3 cells/HPF; P<0.001) and in the Sd area (23 ± 3 cells/HPF; P<0.001; Figure 5A and 5B).

Conclusively with results for CD20^+^ cells, patient survival was significantly increased in case of high CD79a^+^ cell numbers in the Im region (P=0.0135; Figure 5D) when compared to patients with low CD79a^+^ cell infiltration. For the Tu area (P=0.1795; Figure 5C) and in the Sn region (P=0.7904; Figure 5D) no survival differences based on CD79a^+^ cell infiltration were detected.

In concordance with the results for CD20^+^ cells, high or low infiltration by CD79a^+^ cells in the Im around the tumor also showed no correlation with clinical factors like gender, age, T stage, bridging therapy and also not with presence of hepatitis B or C. Comparable to the results for CD20^+^ cells, there was a significant correlation of cirrhosis with higher levels of infiltrating CD79a^+^ cells in the infiltration margin (Supplementary Table 2).

Additionally, CD79a^+^ cells in the Tu and the Im region correlated with CD8^+^ cells in the Tu and Im area (P<0.001; Supplementary Figure 1).

### Expression of immunoglobulins in tumor and infiltrative margin correlates with B-cell expression in patients after HCC resection

**Figure 6:**
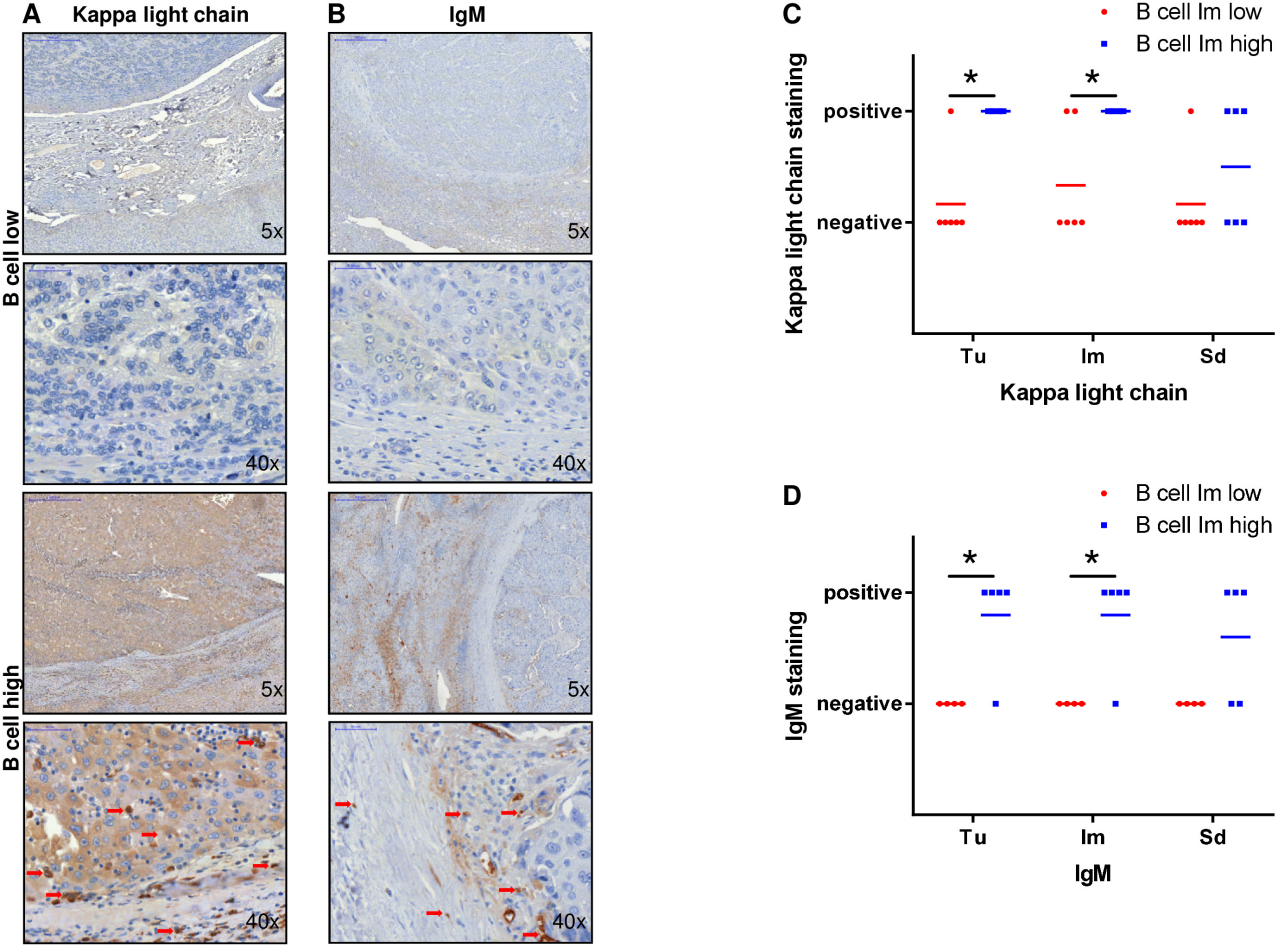
Expression of immunoglobulin fragments in tumor and infiltrative margin correlates with B-cell expression in patients after HCC resection **(A** and **B)** Patient samples with high B-cell numbers showed strong positivity for Kappa light chain and IgM in Tu and Im areas in contrast to patients with low B-cell numbers where no expression of these immunoglobulin fragments was detected. **(C)** Kappa light chain expression of patients with high numbers of B cells in the infiltrative margin was significantly higher in Tu (P=0.0005) and Im area (P=0.0101) when compared to patients with low B-cell numbers without differences in the Sd region (P=0.2596). **(D)** IgM expression of patients with high numbers of B cells in the infiltrative margin was significantly higher in Tu (P=0.0096) and Im area (P=0.0096) when compared to patients with low B-cell numbers without differences in the Sd region (P=0.0676).

Since gene expression analysis has demonstrated the beneficial effect of high expression of immunoglobulin fragments for HCC outcome, we examined if high expression of immunoglobulins on protein level was also associated with high B-cell numbers in the respective tissue. Therefore, patient samples with high and low numbers of B cells in the infiltrative margin were immunostained for Kappa light chain and IgM. Indeed, patients with high B-cell numbers showed strong positivity for Kappa light chain (Figure 6A) and IgM (Figure 6B) in Tu and Im areas in contrast to patients with low B-cell numbers where no expression of these immunoglobulin fragments was detected. This Kappa light chain expression of patients with high numbers of B cells was significantly higher in Tu (P<0.001) and Im area (P=0.0101) when compared to patients with low B-cell numbers in the infiltrative margin (Figure 6C). No difference between these patient groups was detected in the Sd region (P=0.2596). Analogously, IgM expression of patients with high numbers of B cells in the infiltrative margin was significantly higher in Tu (P=0.0096) and Im area (P=0.0096) when compared to patients with low B-cell numbers in the infiltrative margin (Figure 6D). Again, no difference between these patient groups was detected in the Sd region (P=0.0676).

## DISCUSSION

Recently there have been contradictory reports about B-cell effects on HCC outcome.[9–16] Especially tumor infiltration by atypical memory B cells, that are CD20^+^IgD^-^IgG^+^CD27^-^CD38^-^ were significantly associated with improved overall and recurrence-free survival of HCC patients [9]. This antitumor potential is thought to be due to more potent antigen presentation to and activation of T cells, especially CD8^+^ cells in the tumor microenvironment [9, 21]. Contrarily, CXCR3^+^ or regulatory B-cell subsets were shown to significantly correlate with early recurrence and negative patient outcome [12, 13]. These B cells supposedly induce protumorigenic activity of macrophages or directly interact with tumor cells by CD40/CD154 signaling pathway [12, 13]. Generally, B-cell effects on HCCs in these studies with conflicting results are thought to be due to changes in T-cell activity and micromilieu.

This study demonstrates for the first time that B cells are beneficial for HCC patients potentially through direct antitumor effects by secreted immunoglobulins. More specifically, it was demonstrated using a large-scale unsupervised oncogenetic microarray database that immunoglobulins and immunoglobulin fragments were associated with positive HCC outcome. The fact that overexpression of immunoglobulins positively influence HCC patient survival is confirmed in a subcohort of this database and also in two additional independent databases of HCC patients. Little is known about effects of immunoglobulin on HCC promotion. It was reported that HCC patients in comparison to healthy patients and patients with liver cirrhosis without HCC exhibit higher levels of IgG1 and IgM but lower concentrations of IgG2 [22]. Another study showed a trend but no statistical significance to prolonged survival of patients with HCC in case of high IgA and IgG serum levels [23]. Similarly, high total IgG antibody titers against polymorphic epithelial mucin, one of the most specific tumor-associated antigen in patients with breast cancer were correlated with better survival of breast cancer patients, while IgG subclasses that showed statistical significance for survival could not be dissected [24].

Additionally, these positive effects of immunoglobulin overexpression and coregulation in our study were accompanied by high numbers of B cells in the infiltrative margin. This corroborates the hypothesis that these immunoglobulins secreted by B cells have direct antitumor effects. This direct antitumor activity of B cells by immunoglobulin secretion has been reported previously in experimental models [25, 26]. Especially, in combination with IL-17A administration B cells showed enhanced tumor killing capability by promoted B-cell migration and higher production of immunoglobulins [27].

In this present study it is further demonstrated, that high B-cell numbers in the infiltrative margin are associated with improved HCC patient survival. In detail, high numbers of CD20^+^ and CD79a^+^ cells in the infiltrative margin but not in the tumor area and in the liver periphery showed this positive effect on patient outcome after tumor surgery. This confirms previous results from Shi et al. who have also shown that CD20^+^ B cells that infiltrate the infiltrative margin and display an atypical memory phenotype correlate with favorable prognosis in HCC patients [9]. Similarly to these report in HCC, Nielsen et al. have described this beneficial effect of CD27^-^ atypical memory B cells in ovarian cancer [11]. Unlike these two studies that concluded that these antitumor effects are mostly due to changes in T-cell tumoricidal activity our study for the first time additionally demonstrates the importance of changes in immunoglobulin levels for antitumor activity of B cells [9, 11].

The retrospective nature of this study is a possible limitation. However, the results that are generated from 3 independent patient cohorts containing a total of 2519 patients are conclusive. Further, the immunological and genetical factors that in this study were shown to significantly influence patient survival fundamentally extends the limited knowledge about B-cell effects on survival of HCC patients.

In conclusion, this study demonstrates that infiltration of HCCs by high numbers of B cells is associated with prolonged overall survival of these patients. This B-cell infiltration induces a distinct B-cell like immunoglobulin profile of HCCs that was identified to go along with better patient outcome. We suggest that B cells contribute to local tumor control by secreting increased levels of immunoglobulins with antitumor activity.

## MATERIALS AND METHODS

### Study setting

This study was performed at the University Medical Center of Regensburg, Germany and was approved by the local ethics committee (Nr. 12-101-0009).

### Bioinformatics gene expression analysis

The ‘GSE2109’ dataset was obtained from NCBI’s Gene Expression Omnibus 39 via the Bioconductor package ‘GEOquery’ 40 as described previously [17]. Briefly, further analysis was performed using R (http://r-project.org). According to the experimental description, signal values of the probe set were summarized using the Microarray Suite 5.0 (MAS5) and normalized. After downloading and combining the data into a single expression set, the expression data were transformed for each array via the Z-score [18]. Gene-centered information was obtained by summarizing and averaging the expressions of all gene-specific spots per array as described by the annotation GPL570 and documented in the Gene Expression Omnibus. Highly correlated gene expressions were detected by Pearson’s correlation coefficient (CC). Genes with a CC of |CC| > 0.8 were used for further analysis. For hierarchical clustering, distances between genes within the reduced dataset were calculated with Pearson’s CC and transformed through CC = 1 − |CC|, to be used for hierarchical clustering. Complete clustering was applied to transformed distances. To estimate the ideal number of clusters, the KL index and the C index were applied to the clustering result as described previously [17].

### Data analysis for independent validation set

The survival analysis is performed on ´GSE14520` provided by NCBI-GEO [19]. Therefore, the raw data is RMA-normalized per array-platform (Affymetrix Human Genome U133A 2.0, Affymetrix HT Human Genome U133 Array). Due to the same feature annotation on both platforms the annotation of hgu133a2 is used. Gene-centered information was obtained by summarizing and averaging the expressions of all gene-specific spots per array as described by the annotation of hgu133a2. After that, quantile normalizations over both platforms are applied due to the differences between both datasets. The expression values of this dataset were reduced to the cut-set of genes between the clusters identified in the gene expression analysis of ‘GSE2109’. The remaining expression matrix was centered using the mean of the respective gene and normalized via the standard deviation. Next, the expression matrix was centered using the mean of the respective array and normalized via the standard deviation. Then, arrays were grouped using hierarchical clustering. Expression values of each gene are depicted as density and boxplot, differences are described via t-test, wilcox test, fold change and log fold change.

### Patient selection and data collection for immunohistological confirmation set

Patients with HCC (confirmed by histology) who underwent liver surgery for this diagnosis at the University Medical Center of Regensburg between 2004 and 2011 were identified using the hospital computer database. Only patients receiving primary liver resection with curative intent were included. If there was not enough tissue for histological evaluation, patients were excluded from this study. General patient information, preoperative neoadjuvant therapies, TNM classification and histological data on the HCC were obtained from the routine pathology report and the hospital computer database. Survival data were collected using the database of the Tumorzentrum Regensburg (regional tumor center of East Bavaria), Germany.

### Immunohistological analysis

Tissue cross sections were stained for CD20, CD79a, Kappa light chain, IgM and H&E (details in Supplementary Materials). Digital images of the HCCs were obtained at 5x and 20x magnification on light microscopy (Carl Zeiss MicroImaging GmbH, Jena, Germany).

Numbers of CD20^+^ and CD79a^+^ cells were manually counted in 3 randomly selected areas (20x magnification) and the mean was taken separately for tumor region (Tu), infiltrative margin (Im) and distant Stroma (Sd) using ImageJ 1.45s software (Wayne Rasband, National Institutes of Health, USA; example in Supplementary Materials). Im was defined as area within one field of view (20x) next to the tumor and Sd was defined as outmost area of the specimen in a region that was tumor-free. Kappa light chain and IgM staining was evaluated as positive or negative in 3 randomly selected areas (40x magnification).

For survival analysis, patients were divided in two groups depending on numbers of CD20^+^ and CD79a^+^ cells in the different areas. Cell numbers larger than the respective mean were defined as “high”, smaller as “low”.

### Statistical analysis

Kaplan-Meier graphs were calculated for survival analyses. Group comparisons were made using log-rank test. Patient data are presented as median with interquartile range (IQR) or as N with percentages. Cell numbers are described as mean ± SEM. To evaluate correlations with cellular expression profiles of HCCs, Pearson`s correlation, and for continuous variables the Mann-Whitney test was used. The level of significance was set at a probability of P<0.05. All histological evaluations were performed independently in a blinded fashion by 2 examiners. In case of divergent evaluation, a consensus was achieved reevaluating the cross sections together. To explore reproducibility after some time random samples were drawn and reevaluated. In all histological evaluations the concordance rate between the observers and the reproducibility of the results was >95%.

## SUPPLEMENTARY MATERIALS FIGURE AND TABLES


