# Supplementary material for: Tumor-infiltrating B cells producing antitumor active immunoglobulins in resected HCC prolong patient survival
Source: Oncotarget. 2017 Aug 9;8(41):71002–11. doi: 10.18632/oncotarget.20238 (PMC5642613; doi:10.18632/oncotarget.20238)
Supplement: Supplementary file 1 [file oncotarget-08-71002-s001.pdf]

Tumor-infiltrating B cells producing antitumor active immunoglobulins in resected HCC prolong patient survival

SUPPLEMENTARY MATERIALS

Histological methods

Specimens were fixed in 4% paraformaldehyde during operations and then embedded in paraffin. 2µm tissue cross sections were first deparaffinized and then automated stained with a Ventana BenchMark Ultra IHC/ISH Staining Module (Ventana Medical Systems, Inc., Tucson, Arizona, USA) according to the manufacturers staining procedures. As primary antibodies a mouse monoclonal anti-CD20 (REF 760-2537; 1:2; Ventana Medical Systems), a rabbit monoclonal anti-CD79a (REF 790-4432; 1:2; Ventana Medical Systems), a mouse monoclonal anti-Kappa light chain (ab1050; 1:50; Abcam, Cambridge, UK), and a rabbit monoclonal anti-IgM (ab134159; 1:50; Abcam) antibody were used. Streptavidin-HRP reagent (ImmunoBioScience, Everett, Washington, USA) was then applied for 15 minutes, followed by 3,3'-diamino-benzidine tetrahydrochlorhydrate (Merck, Darmstadt, Germany) for 5 minutes. The slides were counterstained with hematoxylin. Further, a H&E staining was performed.

Manual cell count using imageJ 1.45s software (wayne rasband, national institutes of health, USA)

For each patient numbers of $CD20^{+}$ and $CD79a^{+}$ cells were manually counted in 3 randomly selected areas (20x magnification) and the mean was taken separately for tumor region (Tu), infiltrative margin (Im) and distant Stroma (Sd). The software marks the counted cells to enable correct results without double counting.

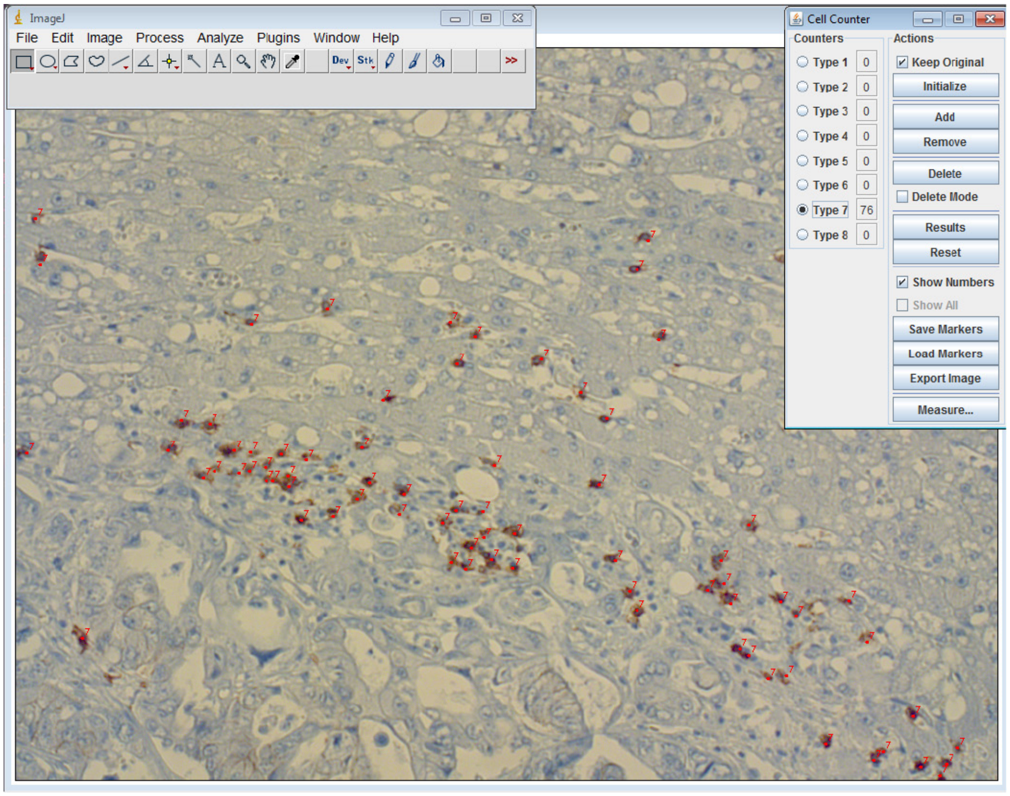

Correlations

		CD20Tu	CD20Im	CD79aTu	CD79aIm	IL33T	IL33Im	CD8T	CD8Im
CD20Tu	Pearson Correlation	1	,154	,639**	,315**	-,039	-,028	,432**	,129
	Sig. (2-tailed)		,106	,000	,001	,691	,774	,000	,181
	N	111	111	111	111	109	109	109	109
CD20Im	Pearson Correlation	,154	1	,121	,407**	,044	,079	,111	,042
	Sig. (2-tailed)	,106		,204	,000	,647	,412	,250	,663
	N	111	111	111	111	109	109	109	109
CD79aTu	Pearson Correlation	,639**	,121	1	,567**	-,112	-,049	,559**	,245*
	Sig. (2-tailed)	,000	,204		,000	,247	,616	,000	,010
	N	111	111	111	111	109	109	109	109
CD79aIm	Pearson Correlation	,315**	,407**	,567**	1	-,083	-,033	,334**	,380**
	Sig. (2-tailed)	,001	,000	,000		,392	,731	,000	,000
	N	111	111	111	111	109	109	109	109
IL33T	Pearson Correlation	-,039	,044	-,112	-,083	1	,318**	,015	,036
	Sig. (2-tailed)	,691	,647	,247	,392		,000	,876	,702
	N	109	109	109	109	117	117	117	117
IL33Im	Pearson Correlation	-,028	,079	-,049	-,033	,318**	1	,237*	,275**
	Sig. (2-tailed)	,774	,412	,616	,731	,000		,010	,003
	N	109	109	109	109	117	117	117	117
CD8T	Pearson Correlation	,432**	,111	,559**	,334**	,015	,237*	1	,453**
	Sig. (2-tailed)	,000	,250	,000	,000	,876	,010		,000
	N	109	109	109	109	117	117	117	117
CD8Im	Pearson Correlation	,129	,042	,245*	,380**	,036	,275**	,453**	1
	Sig. (2-tailed)	,181	,663	,010	,000	,702	,003	,000	
	N	109	109	109	109	117	117	117	117

**. Correlation is significant at the 0.01 level (2-tailed).

*. Correlation is significant at the 0.05 level (2-tailed).

Supplementary Figure 1: Correlation of CD20, CD79a, IL-33 and CD8 positive cells separately for the investigated sample areas. Tu: tumor, Im: infiltrative margin.

Supplementary Table 1: Differences in clinical parameters with regards to CD20^{+} cell infiltration

Clinical parameters	CD20 Im low	CD20 Im high	P-value
		Number or mean	
Gender			
Male / female	51 / 19	36 / 5	0.094
Age (y)	63 ± 1	64 ± 2	0.670
Hepatitis			
None / B / C	61 / 4 / 5	30 / 2 / 9	0.076
T			
1 / 2 / 3 / 4	23 / 22 / 22 / 3	15 / 15 / 10 / 1	0.801
Bridging			
None / TACE / RFA	60 / 5 / 1	40 / 0 / 1	0.141
Cirrhosis No/Yes	36/34	9/32	0.003

Values expressed as mean ± SEM or as number; Im: infiltrative margin; *P<.05* Student´s t -test.

Supplementary Table 2: Differences in clinical parameters with regards to CD79a[+] cell infiltration

Clinical parameters	CD79a Im low	CD79a Im high	P-value
		Number or mean	
Gender			
Male / female	57 / 18	30 / 6	0.465
Age (y)	63 ± 1	63 ± 2	0.899
Hepatitis			
None / B / C	65 / 4 / 6	26 / 2 / 8	0.104
T			
1 / 2 / 3 / 4	25 / 24 / 24 / 2	13 / 13 / 8 / 2	0.671
Bridging			
None / TACE / RFA	67 / 4 / 1	33 / 1 / 1	0.405
Cirrhosis No/Yes	37/38	8/28	0.007

Values expressed as mean ± SEM or as number; Im: infiltrative margin; $*P<.05$ Student´s t -test.
